# Factors associated with the choice of primary care facilities for initial treatment among rural and urban residents in Southwestern China

**DOI:** 10.1371/journal.pone.0211984

**Published:** 2019-02-07

**Authors:** Xiaxia Sun, Hongdao Meng, Zhiqiu Ye, Kyaien O. Conner, Zhanqi Duan, Danping Liu

**Affiliations:** 1 Department of Health and Social Behavior, School of Public Health, Sichuan University, Chengdu, China; 2 School of Aging Studies, College of Behavioral & Community Sciences, University of South Florida, Tampa, Florida, United States of America; 3 Department of Public Health Sciences, University of Rochester Medical Center, Rochester, New York, United States of America; 4 Department of Mental Health Law & Policy, College of Behavioral & Community Sciences, University of South Florida, Tampa, Florida, United States of America; 5 Health and Family Planning Information Centre of Sichuan Province, Chengdu, China; University Lyon 1 Faculty of Dental Medicine, FRANCE

## Abstract

**Objective:**

To explore influential factors contributing to the choice of primary care facilities (PCFs) for the initial treatment among rural and urban residents in Southwestern China.

**Methods:**

A face-to-face survey was conducted on a multistage stratified random sample of 456 rural and 459 urban residents in Sichuan Province from January to August in 2014. A structured questionnaire was used to collect data on residents’ characteristics, provider of initial treatment and principal reason for the choice. Multivariate logistic regression was performed to identify factors associated with choosing PCFs for the initial treatment.

**Results:**

The result showed that 65.4% of the rural residents and 50.5% of the urban residents chose PCFs as their initial contact for medical care. Among both rural and urban residents, the principal reason for choosing medical institutions for the initial treatment was convenience (42.3% versus 40.5%, respectively), followed by high quality of medical care (26.5% versus 29.4%, respectively). Compared to rural residents, urban residents were more likely to value trust in doctors and high quality of medical care but were less likely to value the insurance designation status of the facilities. Logistic regression analysis showed that both rural and urban residents were less likely to choose PCFs for the initial treatment if they lived more than 15 minutes (by walk) from the nearest facilities (rural: OR = 0.15, 95%CI = 0.09–0.26; urban: OR = 0.19, 95%CI = 0.10–0.36), had fair (rural: OR = 0.49, 95%CI = 0.26–0.92; urban: OR = 0.31, 95%CI = 0.15–0.64) or poor (rural: OR = 0.14, 95%CI = 0.07–0.30; urban: OR = 0.22, 95%CI = 0.11–0.44) self-reported health status. Among rural residents, attending college or higher education (OR = 0.21, 95%CI = 0.08–0.59), being retired (OR = 0.90, 95%CI = 0.44–1.84) and earning a per capita annual income of household of 10,000–29,999 (OR = 0.24, 95%CI = 0.11–0.52) and 30,000–49,999 (OR = 0.26, 95%CI = 0.07–0.92) were associated with lower rates of seeking care at PCFs.

**Conclusion:**

Efforts should be made to improve the accessibility of PCFs and to upgrade the services capability of PCFs both in rural and urban areas in China. At the same time, resources should be prioritized to residents with poorer self-reported health status, and rural residents who retire or have better education and higher income levels should be taken into account.

## Introduction

“Significant challenges plague the health care system in China with one of the most common complaints being that it is too difficult and too expensive to see a doctor”[1]. One of the main reasons may be that residents prefer to seek care in second or tertiary hospitals rather than in primary care facilities (PCFs), despite that PCFs provide care that is usually more accessible and less costly. In the health system in China, PCFs play an important role in the management of acute and chronic conditions and the reduction of disease burdens[2, 3]. To improve the utilization of PCFs, the Chinese government proposed to establish a hierarchical diagnosis and treatment system in a new round of medical reform in 2009.

In the hierarchical diagnosis and treatment system, PCFs serve as the point for initial triage by treating acute illnesses and managing chronic conditions as well as referring patients up the hierarchy when necessary[4, 5]. The policy recommends that residents should receive initial treatment at PCFs, which consists of community health centers (CHCs) and community health stations (CHSs) in urban areas, and township hospitals and village clinics in rural areas[6]. Small private outpatient clinics and pharmacy clinics in urban and rural areas are also considered PCFs. The policy is similar to the “gatekeeping system” or “family doctor system” that has played a very important role in the health systems in many countries, such as Germany[7], the United Kingdom[8], Spain[9], Switzerland and the Netherlands[10].

To implement this policy, the Chinese government has made significant efforts in financing and training. First, the government has increased the financial investment in PCFs. For example, the proportion of government financial input increased from 20.7% in 2008 to 46.8% in 2011 for CHCs and from 17.5% to 37.9% for township hospitals[11]. As a result, the number of PCFs has increased rapidly from 858,015 in 2009 to 926,518 in 2017[12]. Second, the government also attracted and retained general practitioners (GPs) of high quality in PCFs. In 2013, about 37.1% of CHCs’ health practitioners had a bachelor’s degree or above, in contrast with 21.9% in 2005[13]. Additionally, a differential reimbursement system was established, which offers payment incentives for services delivered at PCFs.

However, residents’ health-seeking behaviors remain largely unchanged, particularly among residents in the urban areas. According to the fifth National Health Service Survey in 2013, 19.9% and 34.8% of patients opted for care provided by higher-tier hospitals in rural and urban areas, respectively[14]. Previous researches have showed that a considerable number of residents still choose higher-tier hospitals for the initial treatment[9, 15], even for common conditions such as chronic conditions[16, 17]. Existing reasons for residents bypassing local PCFs[18] are related to residents' distrust[19], dissatisfaction[20], concerns about the lack of medical equipment[21] and so on.

Though researches have examined the factors contributing to residents’ decision to use PCFs to meet their primary care needs, only patients who received care at PCFs were included[22, 23]. Given the urban-rural disparities in economic development and supply of health care services during the rapid economic growth in recent decades, it is important to examine the urban-rural differences in primary care seeking behavior[24]. Compared to urban residents, rural residents may opt to receive care at PCFs for different reasons due to financial constraints, limited access to higher-tier hospitals[25, 26] and so on, which may have different policy implications for strategies to promote the utilization of PCFs. Previous studies only examined rural or urban residents’ primary care seeking behavior alone[23, 27]. Additionally, little is known about the differences in the utilization rates of PCFs between rural and urban residents. The objective of this study was to examine factors associated with the choice of PCFs for initial treatment between rural and urban residents. Findings from this study may have important implications for strategies to improve the utilization of PCFs and the patient flows between PCFs and higher-tier hospitals in both rural and urban areas.

## Materials and methods

### Ethics statement

This study protocol was approved by the Institutional Review Board of School of Public Health, Sichuan University. All participants read a statement that explained the purpose of the survey and written informed consents have been received before being involved in the investigation.

### Study setting

This cross-sectional study was conducted by trained investigators (medical and public health students and community volunteers) in Sichuan province, from January to August in 2014. Sichuan Province is the largest province in Southwestern China (by population) with lower economy level and poorer medical and health services than the central or eastern region of China generally. In 2017, approximately 49.2% of the population of Sichuan province resided in urban areas, as compared to 57.4% for China overall[12].

### Participants

**Fig 1:**
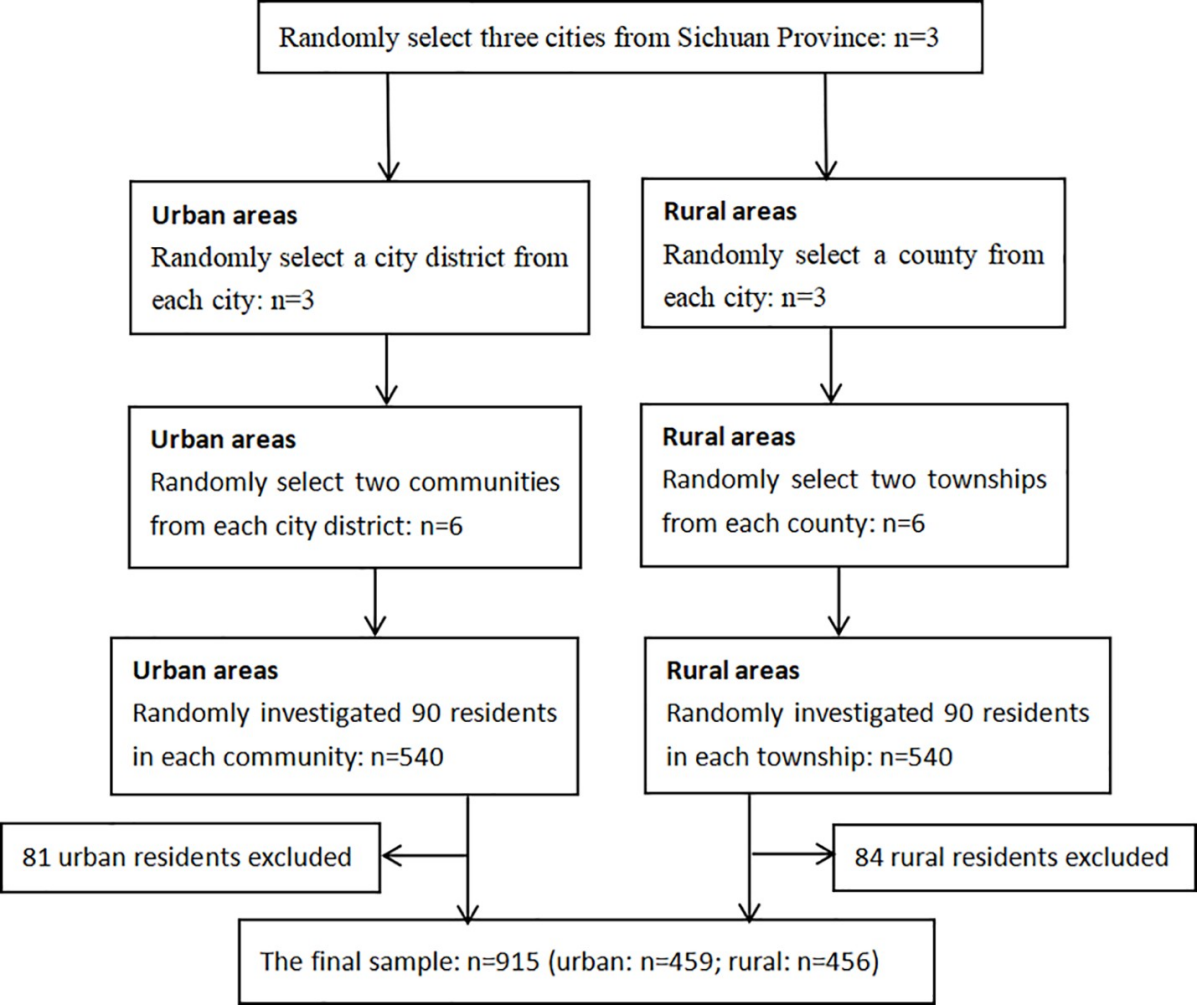
Sampling flow-chart.

A multistage stratified random sampling method was used. In the first stage, three cities in Sichuan Province were randomly selected. In the second stage, a city district and a county were randomly selected from each city. In the third stage, two communities or townships were randomly selected from each city district or county. In the fourth stage, we randomly selected 90 residents in each community or township and asked about their choices of PCFs as the initial contact for medical care during their latest illness episode (**Fig 1**). Residents who were 18 years old and above and had resided in the community/town for at least 6 months were eligible to participate.

The investigators took an average of 15 minutes to interview each participant. Questionnaires were checked by investigators immediately after the survey for completeness. A total of 1,080 residents were interviewed (540 urban and 540 rural). Because the present survey focused on residents’ choices of PCFs for the first treatment during the latest illness episode, only those who sought medical treatment were used in the analysis and 165 residents (81 urban and 84 rural) were excluded for not seeking medical treatment during the latest illness episode. The final sample included 915 residents (459 urban and 456 rural).

### Measures

Respondent characteristics, provider of initial treatment and principal reason for the choice were collected.

#### Respondent characteristics

Respondent characteristics were collected, including socio-demographic characteristics, walking time (minutes) from home to the nearest PCFs and self-reported health status. Socio-demographic characteristics included age, gender, marital status, employment status, education, status of medical insurance, per capita annual income of household and individual annual income.

#### Provider of initial treatment and principal reason for the choice

The places of care for the initial treatment were categorized into two basic types: PCFs and higher-tier hospitals. For residents who chose to receive initial treatment at medical institutions, we asked them to select principal reason for the choice, including convenience, whether they felt that the charges were reasonable, quality of care, trust in doctors, good patient-doctor communication, prior experience with the doctors, and the medical insurance designation status of the facility.

### Statistical analysis

Data were entered using the Epidata 3.1 database and were analyzed using the IBM SPSS version 23.0. We reported means and SDs for continuous variables and percentages for categorical variables. Pearson’s Chi-square tests or Fisher’s exact test was used to assess the differences in categorical variables. We conducted multivariable logistic regression models to identify the factors associated with rural or urban residents’ decisions about whether to seek care at PCFs for the initial treatment. The dependent variable was whether the residents used PCFs (0 for no and 1 for yes). The odds ratio (OR) was reported along with 95% confidence interval (CI). Results with a p-value of <0.05 were considered statistically significant.

## Results

### Characteristics of the urban and rural residents

Results of individual characteristics of the 915 residents (459 in urban, 456 in rural) who sought medical treatment in the latest illness episode are shown in Table 1. Most of the residents were female (56.0% in urban, 61.4% in rural), married (80.6% in urban, 82.5% in rural) and had medical insurance (94.6% in urban, 91.4% in rural). Approximately 40.1% of the residents in the urban areas were in the 65 and above age group, whereas only 4.4% of them aged 18 to 24 years. Residents in the rural areas were younger and demonstrated larger variations (**Table 1**). Most of the residents received an education of less than high or vocational school (61.9% in urban, 79.7% in rural, p<0.001). More than half (60.1%) of the respondents in the rural areas were employed, compared to 25.1% of the respondents in the urban areas. In contrast, only 12.5% of the residents in the rural areas were retired, compared to 43.6% of those in the urban areas. For both rural and urban residents, the greatest proportion of residents had a per capita annual income of household between 10,000 to 29,999 yuan (RMB). Nevertheless, a considerable number of residents had a per capita annual income of household less than 5000 yuan (RMB), particularly among those in the rural areas (6.5% in urban, 25.0% in rural, p<0.001). Results were similar for the distribution of the variable of individual annual income. More than half of the urban residents (52.5%) reported health status as poor, whereas 23.7% of the rural residents reported as poor. Most residents need less than 15 minutes from home to the nearest PCFs (83.0% in urban, 71.5% in rural, p<0.001).

**Table 1:** Characteristics of the participants.

Characteristics	Urban Residents		Rural Residents		*χ*^*2*^	*P*-value
	n	%	n	%		
Gender					2.764	0.096
Male	202	44.0	176	38.6		
Female	257	56.0	280	61.4		
Age group					74.485	<0.001
18–24 years	20	4.4	42	9.2		
25–34 years	30	6.5	46	10.1		
35–44 years	24	5.2	82	18.0		
45–54 years	64	13.9	82	18.0		
55–64 years	137	29.8	104	22.8		
≥65 years	184	40.1	100	21.9		
Marital status					0.518	0.472
Single	89	19.4	80	17.5		
Married	370	80.6	376	82.5		
Employment status					145.892	<0.001
Currently employed	115	25.1	274	60.1		
Retired	200	43.6	57	12.5		
Unemployed	144	31.4	125	27.4		
Educational level					35.750	<0.001
Illiteracy and primary school	172	37.5	210	46.1		
Middle school	112	24.4	153	33.6		
High or vocational school	76	16.6	45	9.9		
College and above	99	21.6	48	10.5		
Medical insurance					3.392	0.066
Yes	434	94.6	417	91.4		
No	25	5.4	39	8.6		
Per capita annual income of household (RMB), yuan					198.541	<0.001
<5000	30	6.5	114	25.0		
5000–9999	48	10.5	129	28.3		
10000–29999	171	37.3	170	37.3		
30000–49999	92	20.0	26	5.7		
≥50000	118	25.7	17	3.7		
Individual annual income (RMB), yuan					74.915	<0.001
<5000	65	14.2	144	31.6		
5000–9999	56	12.2	97	21.3		
10000–29999	239	52.1	171	37.5		
30000–49999	65	14.2	34	7.5		
≥50000	34	7.4	10	2.2		
Walking time from home to the nearest PCFs					17.269	<0.001
≤15 min	381	83.0	326	71.5		
>15 min	78	17.0	130	28.5		
Self-reported health status					88.352	<0.001
Good	73	15.9	159	34.9		
Fair	145	31.6	189	41.4		
Poor	241	52.5	108	23.7		

### Residents’ choices of PCFs for the initial treatment

**Table 2** describes the urban and rural residents’ choices of medical institutions for the initial treatment during their latest episode of illness among those who had sought medical treatment (n = 915). Overall, 530 residents (57.9%) responded that they sought care at PCFs for the initial treatment. More than half of the rural residents (65.4%) reported receiving care at PCFs, compared to 50.5% of those in the urban areas (*p*<0.001).

**Table 2:** Urban and rural residents’ choices of PCFs for the initial treatment (n = 915).

Choices	Urban Residents		Rural Residents		*χ*^*2*^	*p*-value
	n	%	n	%		
PCFs	232	50.5	298	65.4	20.575	<0.001
Secondary or tertiary hospitals	227	49.5	158	34.6		

### Residents’ principal reason for choosing medical institutions for the initial treatment

**Table 3** shows urban and rural residents’ principal reason for choosing medical institutions for the initial treatment (n = 915). The results showed that many residents, both in urban (40.5%) and rural (42.3%) areas, chose medical institutions for the initial treatment for the convenience of seeking healthcare. Also, 29.4% of the urban residents and 26.5% of the rural residents described good services quality as their principal reason for choosing medical institutions for the initial treatment. There were significant differences between the urban and the rural residents in the role of the quality of services, trust in doctors and medical insurance designated status of hospitals (*p*<0.05). Specifically, compared to residents in the rural areas, residents in the urban areas were more likely to report quality of services and trust in doctors rather than the facilities’ status of insurance designation as the principal reason to select a medical institution for the initial treatment (*p*<0.05, Table 3).

**Table 3:** Urban and rural residents’ principal reason for choosing medical institutions for the initial treatment (n = 915).

	Urban Residents		Rural Residents		*χ*^*2*^	*p*-value
	N	(%)	N	(%)		
Convenience					0.306	0.580
Yes	186	40.5	193	42.3		
No	273	59.5	263	57.7		
Reasonable charges					2.231	0.135
Yes	45	9.8	59	12.9		
No	414	90.2	397	87.1		
Good quality of care					4.138	**0.042**
Yes	135	29.4	121	26.5		
No	276	60.1	335	73.5		
Trust in doctors					*	**0.002**
Yes	45	9.8	20	4.4		
No	414	90.2	436	95.6		
Medical insurance designated facility					*	**0.006**
Yes	12	2.6	29	6.4		
No	447	97.4	427	93.6		
Good patient-doctor communication					*	0.694
Yes	33	7.2	29	6.4		
No	426	92.8	427	93.6		
Prior experience with the doctors					*	0.505
Yes	3	0.7	5	1.1		
No	456	99.3	451	98.9		

* Fisher’s exact test.

### Factors influencing residents’ choices for PCFs for the initial treatment

**Table 4** shows the results of the multivariable logistic regression models predicting the likelihood of selecting PCFs for the initial treatment, stratified by rural-urban location. Urban residents who lived more than 15 minutes away from the nearest PCFs were less likely to seek care at PCFs (OR = 0.19, 95%CI = 0.10–0.36). Urban residents who rated health status as fair (OR = 0.31, 95%CI = 0.15–0.64) and poor (OR = 0.22, 95%CI = 0.11–0.44) were less likely to seek care at PCFs when compared with those who rated health status as good.

**Table 4:** Multivariable logistic regression analysis of factors associated with the urban and rural residents’ choices for PCFs for the initial treatment (n = 915).

Variables	Reference Category	Urban Residents		Rural Residents	
		OR	95% CI	OR	95% CI
Constant		5.34	-	225.94	-
Gender	Female	1.36	0.87–2.13	1.17	0.67–2.05
Age	18–24 years				
25–34 years		1.77	0.47–6.61	0.98	0.29–3.32
35–44 years		4.08	0.96–17.34	1.14	0.37–3.51
45–54 years		1.76	0.51–6.09	1.77	0.56–5.64
55–64 years		1.63	0.45–5.94	1.28	0.40–4.10
≥65 years		0.87	0.24–3.18	0.64	0.20–2.01
Marital status	Single				
Employment status	Unemployed	0.82	0.37–1.83	1.33	0.69–2.57
Currently employed		0.97	0.54–1.75	0.21	0.09–0.52
Retired		1.29	0.72–2.31	0.90*	0.44–1.84
Education level	Illiteracy and primary school				
Middle school		1.00	0.56–1.79	1.27	0.66–2.44
High or vocational school		0.64	0.31–1.29	2.00	0.72–5.54
College and above		0.73	0.34–1.58	0.21*	0.08–0.59
Medical insurance	Yes	1.30	0.50–3.35	1.08	0.42–2.79
Individual annual income (RMB), yuan	<5000				
5000–9999		0.67	0.26–1.71	0.58	0.27–1.26
10000–29999		0.86	0.39–1.90	0.79	0.37–1.68
30000–49999		0.67	0.24–1.82	0.74	0.21–2.57
≥50000		1.57	0.48–5.09	0.31	0.06–1.61
Per capita annual income of household (RMB), yuan	<5000				
5000–9999		1.71	0.53–5.51	0.61	0.28–1.35
10000–29999		2.59	0.90–7.41	0.24***	0.11–0.52
30000–49999		1.46	0.48–4.50	0.26*	0.07–0.92
≥50000		0.93	0.28–3.02	0.70	0.13–3.89
Walking time from home to the nearest PCFs	≤15 minutes	0.19***	0.10–0.36	0.15***	0.09–0.26
Self-reported health status	Good				
Fair		0.31***	0.15–0.64	0.49*	0.26–0.92
Poor		0.22***	0.11–0.44	0.14***	0.07–0.30

* significant at *p*<0.05; ** significant at *p*<0.01

*** significant at *p*<0.001.

Abbreviation: PCFs, primary care facilities; OR, odds ratio; CI, confidence interval.

Similar to urban residents, rural residents who lived more than 15 minutes away from the nearest PCFs were less likely to choose PCFs for the initial treatment (OR = 0.15, 95%CI = 0.09–0.26). Rural residents who rated health status as fair (OR = 0.49, 95%CI = 0.26–0.92) and poor (OR = 0.14, 95%CI = 0.07–0.30) were less likely to choose PCFs for the initial treatment when compared with those who rated health status as good. In addition, retired rural residents were less likely to choose PCFs for the initial treatment as compared with unemployed rural residents (OR = 0.90, 95%CI = 0.44–1.84). Rural residents who were educated at college level and above were less likely to choose PCFs for the initial treatment when compared with those educated illiteracy and primary school (OR = 0.21, 95%CI = 0.08–0.59). Rural residents who had a per capita annual income of household of 10,000–29,999 yuan (OR = 0.24, 95%CI = 0.11–0.52) and 30,000–49,999 yuan (OR = 0.26, 95%CI = 0.07–0.92) were less likely to choose PCFs for the initial treatment when compared with those earned less than 5,000 yuan.

## Discussion

This study is the first empirical analysis comparing rural and urban residents’ choices for PCFs as the initial contact for medical care in a random sample of residents from Southwestern China. We identified notable differences in the patterns of and the determinants to the utilization of PCFs for the initial treatment between rural and urban residents.

The overall rate of choosing PCFs for the initial treatment is 57.9%, with a higher rate among rural residents (65.4%) than among urban residents (50.5%) (*p*<0.001). It is still far behind 70% that was recommended by the Chinese government[28]. The rate among urban residents (50.5%) is lower than the results from the previous studies mainly in eastern and central China in which 62.2%-93.3% of urban residents preferred PCFs for the initial treatment[22, 29, 30]. One reason for the differences may be that the previous study samples were recruited from patients receiving care at PCFs who might already have preference for them, while no such bias is expected due to the random sampling of individuals from the communities in this study. Similarly, the utilization rate of PCFs for the initial treatment among rural residents (65.4%) is also lower than the results of a study conducted in rural eastern China by Ye and colleagues (72.6%)[23]. This might be explained by the shortage of medical practitioners[31] and the insufficiency of PCFs’ overall services capability[32] in rural Southwestern China. Previous research has found that patients are generally not satisfied with the primary care services in Western China, particularly in the rural areas[20]. Thus, efforts should be strengthened to improve the utilization rate of PCFs for the initial treatment both in rural and urban areas, particularly in Southwestern China.

The study showed two common principal reasons for choosing medical institutions for the initial treatment between rural and urban residents. Many residents, both in urban (40.5%) and rural areas (42.3%), indicated that they chose medical institutions for the initial treatment because of convenience, which is consistent with previous studies[33, 34]. This is mainly because patients generally prefer to visit medical institutions closer to their homes[35]. In addition, 29.4% of the urban residents and 26.5% of the rural residents rated good quality in medical care as a high priority in their reason for choosing medical institutions for the initial treatment, compared to other aspects of quality such as patient-physician communication. This supports the findings of a previous hospital choice experiment in which quality of care showed a relative higher importance than other factors such as hospital atmosphere and waiting time when choosing a hospital for surgical procedures[36]. This suggests that improving quality of services of PCFs will have a large impact on promoting the utilization rates of PCFs for the initial treatment, which may be particularly important for urban residents as a higher proportion of urban residents reported this factor as the principal reason for choosing medical institutions for the initial treatment. However, currently the service capacity and quality in primary care facilities are lower than that in higher-tier hospitals[37, 38]. Therefore, efforts should be made to upgrade the services capability of PCFs in providing high quality services both in rural and urban areas in China.

The study also showed several important differences in principal reasons for the choice between urban and rural residents. Rural residents were more likely to choose medical institutions for the initial treatment for the reason of medical insurance designated facility when compared to urban residents(*p*<0.001) This might be due to the higher reimbursement rate in PCFs for care delivered for the initial treatment when compared to higher-tier hospitals[39, 40] and rural residents’ lower income than urban residents in China[41].

Urban residents were more likely to select medical institutions for the initial treatment based on trust in the doctors when compared to rural residents. Previous studies have indicated that lack of trust in doctors was a barrier to the utilization for PCFs[15, 42]. In our study, trust appeared to be more important in the decision makings among urban residents. This might have explained the lower utilization rates of PCFs among residents in the urban areas. It is imperative to intensify efforts to improve the service quality of PCFs to increase residents' trust, particularly for residents in the urban areas.

The results of this study identified a group of common characteristics that influence residents’ choices of PCFs for the initial treatment, both in rural and urban areas. Distance to the nearest PCFs was found to be negatively associated with the choice of PCFs among both rural and urban residents, which is consistent with the previous studies[35, 43, 44]. The results suggest that having access to a PCF in the neighborhoods is an important facilitating factor to the use of PCFs. Therefore, efforts focusing on improving the access of PCFs will possibly result in a higher utilization rate of PCFs.

We also find that residents with poorer self-reported health status, both in rural and urban areas, are less likely to utilize PCFs for the initial treatment. The results are consistent with an empirical evidence provided by Tang[45] and other researchers[46, 47]. Self-reported health status is a valid predictor to subsequent morbidity[48] and has been found to be negatively associated with the utilization of health care services[49]. It is a popular believe in China that higher-tier hospitals are of higher quality[50]. Thus, residents with a poorer self-reported health status are more likely to choose higher-tier hospitals over PCFs for primary care. Efforts targeting at residents of poorer health, for example, promising to referring patients with poorer health status to secondary or tertiary hospitals timely, may be effective in promoting the use of PCFs over higher-tier hospitals.

This study also identified several factors that appeared to have a greater impact on the choice of PCFs among rural residents, such as education, employment status and income, three most common socioeconomic status (SES) factors predicting healthcare seeking behavior[51–53]. After controlling for health and demographic characteristics, retired rural residents and those of higher education and higher income levels were less likely to choose PCFs for the initial treatment, which is consistent with prior study[54]. These rural residents may have more resources to manage the higher costs of the services provided at higher-tier medical institutions[18, 27]; well-educated residents have access to higher-tier hospitals more easily[27]. Tai et al. also showed the positive relation between patients’ socioeconomic status and higher-tier medical institutions[55]. This suggests that future efforts to promote the use of PCFs in rural areas should focus on residents with higher SES. The potential mechanism for the relationship between SES and the use of PCFs for the initial treatment is worth extensive inquiry, future studies should explore more about the potential mechanisms for the relationship between SES and the use of PCFs.

This study has several limitations. First, the cross-sectional design precludes us from making causal inferences about the findings. In addition, we did not collect data about quality and patient satisfaction toward the care provided at PCFs, which may also influence the choices of medical institutions for the initial treatment.

## Conclusion

The empirical results show that the rate of seeking care at PCFs for initial treatment remains low in Southwestern China, particularly among residents in the urban areas. Urban and rural residents show two common principal reasons for choosing medical institutions for the initial treatment, including convenience of seeking healthcare and good services quality. However, compared to rural residents, urban residents are more likely to value trust in the doctors and high quality of medical care but are less likely to value the insurance designation of the facilities. The common factors associated with lower utilizations of PCFs for both rural and urban residents include longer distance from home and poorer self-reported health status. Rural residents who have higher SES (i.e. higher income, better education and those who are retired) are less likely to utilize PCFs. The results imply the implementation of the hierarchical diagnosis and treatment system is still far from ideal and that PCFs is still underutilized. Efforts should be made to improve the accessibility of PCFs and to upgrade the services capability of PCFs both in rural and urban areas in China. At the same time, residents with poorer self-reported health status and rural residents who retire or those who have better education and higher income levels should be taken into account. Findings from the research would be benefit for improving the utilization rates of PCFs and rationalizing the patient flows between PCFs and higher-tier hospitals in China, thus helping to solve the problem that it is too difficult and too expensive to see a doctor for residents in China.

## Supporting information

S1 Appendix Questionnaire of the study. (DOCX)

S1 Dataset (SAV)
