# Supplementary material for: Factors associated with the choice of primary care facilities for initial treatment among rural and urban residents in Southwestern China
Source: PLoS One. 2019 Feb 7;14(2):e0211984. doi: 10.1371/journal.pone.0211984 (PMC6366770; doi:10.1371/journal.pone.0211984)
Supplement: S1 Appendix — (DOCX) [file pone.0211984.s001.docx]

**Questionnaire**

**Part 1: Questions on basic information**

1. What is your gender?

1）Man 2）Female

2、Your age： years old（chronological age）

3、Where is your place of residence?

1）In rural 2）In urban

4、What is your marital status?

1）Single 2）Married

5、What is your employment status?

1）Currently employed 2）Retired 3）Unemployed

6、What is your education level?

1）Elementary school or less 2）Middle school

3）High or vocational school 4）College and above

7、Do you have a social medical insurance ?

1）Yes 2）No

8、Your per capita annual income of household (RMB):

1）＜5000 yuan 2）5,000-9,999 yuan 3）10,000-29,999 yuan

4）30,000-4,999 yuan 5）≥50,000 yuan

9、Your individual annual income (RMB):

1）＜5000 yuan 2）5,000-9,999 yuan 3）10,000-29,999 yuan

4）30,000-49,999 yuan 5）≥50,000 yuan

10、How long (minutes) does it take to walk from home to the nearest PCFs?

1）≤15 min 2）＞15 min

11、How is your self-reported health status?

1）Good 2）Fair 3）Poor

**Part 2: Questions on provider of initial treatment and the principal reason for the choice**

12、Did you visit a doctor during the latest illness episode?

1）Yes 2）No (**End the questionnaire**)

13、Which type of medical institutions did you choose as the initial contact for medical care during the latest illness episode?

1）PCFs (CHCs, CHSs, township hospitals, village clinics, small private outpatient clinics and pharmacy clinics)

2）Higher-tier hospitals

14、What is the principal reason for the choice of the medical institutions as the initial contact for medical care?

1）Convenience of seeking health care

2）Reasonable charges

3）Good services quality

4）Trust in doctors

5）Medical insurance designation status of the facility

6）Good patient-doctor communication

7）Prior experience with the doctors

**调查问卷**

**第一部分：基本信息**

1、您的性别：

1）男 2）女

2、您的年龄： 岁（实足年龄）

3、您的居住地是：

1）农村 2）城市

4、您的婚姻状况：

1）未在婚 2）在婚

5、您的就业状况：

1）在业 2）离退休 3）无业或失业（包括学生）

6、您的文化程度：

1）小学及以下 2）初中

3）高中或技校 4）大学及以上

7、您是否参加了社会医疗保险？

1）是 2）否

8、去年一年您的家庭人均年收入大约是多少元？

1）＜5,000 2）5,000-9,999 3）10,000-29,999

4）30,000-49,999 5）≥50,000

9、您最近一年的收入是多少元？

1）＜5,000 2）5,000-9,999 3）10,000-29,999

4）30,000-49,999 5）≥50,000

10、从您家到最近的医疗点步行需要多少分钟？

1）≤15分钟 2）＞15分钟

11、您认为自己的健康状况：

1）好 2）一般 3）差

**第二部分：首诊机构及选择首诊机构的最主要原因**

12、您最近一次生病时是否就诊？

1）就诊 2）未就诊**（结束问卷）**

13、最近一次生病时您最先去的哪里看病（即首诊医疗卫生机构类型）？

1）基层医疗卫生机构（包括：社区卫生服务中心，社区卫生服务站，乡镇卫生院，村卫生室，私人诊所及药店）

2）二、三级医疗卫生机构

14、您选择上述首诊机构的最主要原因是？**（单选）**

1）方便

2）价格合理

3）服务质量高

4）有信赖的医生

5）医疗保险定点单位

6）与医生沟通良好

7）有熟悉的医生
